# Green electrosynthesis of bis(indolyl)methane derivatives in deep eutectic solvents

**DOI:** 10.1186/s13065-024-01245-9

**Published:** 2024-07-27

**Authors:** Mina E. Adly, Amr M. Mahmoud, Hala B. El-Nassan

**Affiliations:** 1 Pharmaceutical Organic Chemistry Department, Faculty of Pharmacy, Cairo University, 33 Kasr El-Aini Street, Cairo, 11562 Egypt; 2 Pharmaceutical Analytical Chemistry Department, Faculty of Pharmacy, Cairo University, Cairo, 11562 Egypt

**Keywords:** Bisindolylmethane, Electrochemical synthesis, Deep eutectic solvents, Green chemistry

## Abstract

In this study, a new green method was developed for the synthesis of bis(indolyl)methane derivatives using electrochemical bisarylation reaction in deep eutectic solvents as a green alternative to traditional solvents and electrolytes. The effects of varying time, current, type of solvent and material of electrodes were all studied. The optimum reaction conditions involved the use of ethylene glycol/choline chloride with a ratio of 2:1 at 80 °C for 45 min. Graphite and platinum were used as cathode and anode, respectively. The newly developed method offered many advantages such as using mild reaction conditions, short reaction time and affording high product yields with a wide range of substituted aromatic aldehydes bearing electron donating or electron withdrawing substituents. In addition, the electrochemical method proved to be more effective than heating in deep eutectic solvents and afforded higher yields of products in shorter reaction time. The mechanism of the electrochemical reaction was proposed and confirmed using the cyclic voltammetry study.

## Introduction

**Fig. 1:**
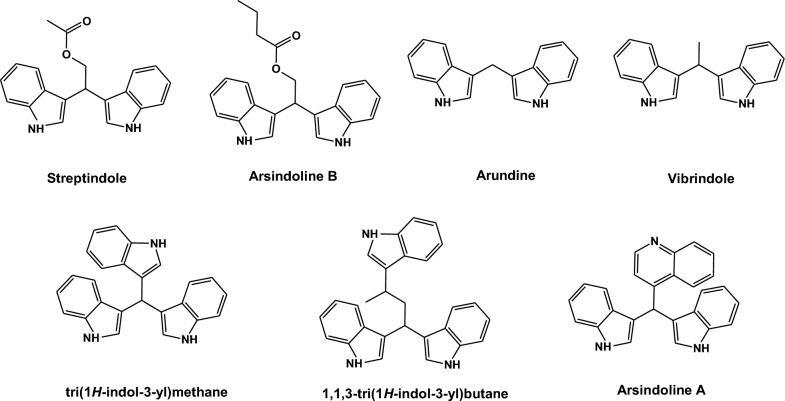
Bis(indolyl)methane derivatives isolated from natural sources

The 3,3ʹ-bis(indolyl)methanes (BIMs) represent an important biologically active scaffold owing to its various biological activities [1, 2]. Many bisindolylmethane alkaloids were isolated from marine natural sources (Fig. 1) [3, 4]. BIM derivatives were reported to exhibit anticancer [5–13], antibacterial [14–16], antifungal, antileishmanial [17], nematocidal activity [18], antioxidant [12, 19], analgesic and anti-inflammatory activities [20, 21]. Additionally, BIMs displayed enzyme inhibitory activity against human carboxylesterase (CES1 and CES2) [22]. The application of BIMs in analytical chemistry involved their use as chemosensors for transition metal cations [23] and for Cu^2+^ ions [24]. The oxidized BIMs could act as selective colorimetric sensors either for fluoride ions in an aprotic solvent or for hydrogen sulfate ions in water [25].

BIMs are prepared by alkylation of indole with aldehyde or ketone. Usually, a Lewis acid or protic acid such as HCl [5] and sulphuric acid [26] are used to catalyze the reaction. However, the use of these acids has many environmental hazards in addition to long reaction times, low yield, and difficult reaction work-up. Therefore, many catalysts were reported in the literature for the synthesis of BIMs aiming to shorten the reaction time and increase the yield with minimal hazard to the environment. Examples included the use of metal salts such as RuCl_3_ [27]; nickel-iodide NiI_2_ [28], hydrated ferric sulfate [29], lithium perchlorate [30], and aluminium triflate [16]. The reaction was catalyzed by some inorganic salts such as NaHSO_3_ [20, 21], KHSO_4_ [23], tetrabutylammonium hydrogen sulfate [31], and boric acid [32]. Other green catalysts used include oxalic acid dihydrate and *N*-cetyl-*N,N,N*-trimethylammonium bromide [5], Meldrum’s acid-induced and FeCl_3_-catalyzed domino reactions [33], *p*-toluene sulfonic acid [14, 19], squaric acid [34] and citric acid [35]. Heterogeneous catalysts like polymer supported dichlorophosphate (PEG-OPOCl_2_) [6], perlite-polyphosphoric acid (EP-PPA) [36], Amberlyst-15 [26, 37], silica supported-boron sulfonic acid [38], Fe-pillared interlayered clay (Fe-PILC) [17] and zeolite [39] were all used to catalyze the reaction.

Moreover, some green chemistry approaches were applied to prepare BIMs. This included the use of visible light for 3–5 h [22], and the use of microwave irradiation for 6–10 min in the presence of surfactant like sodium bis-2-ethyl hexyl sulphosuccinate [40]. The use of ultrasonication was also widely applied for the synthesis of BIMs. Examples included the use of ultrasonication either in acetic acid/water at 40 °C for 2–8 h [18] or in water and dodecylbenzenesulfonic acid catalyst at room temperature for 10–30 min [41]. BIMs were also prepared using the green natural catalyst taurine (NH_2_CH_2_CH_2_SO_3_H) in water under ultrasonic irradiation [42]. The ultrasonic irradiation using *p*-toluene sulfonic acid in acetonitrile was also reported [19]. Biocatalysis of the reaction involved the use of enzymes like α-chymotrypsin [43] and lipase [44].

Ionic liquids can serve as both solvents and catalysts to prepare BIMs [45–48]. Deep eutectic solvents (DESs) are considered a green alternative to ionic liquids. They are formed of H-bond donor and acceptor that interact to produce a low melting eutectic mixture. They are characterized by being nontoxic, easily prepared, recyclable, and eco-friendly [49–55]. The synthesis of BIM derivatives was performed in choline chloride-urea (1:2) at 70 °C for 4 h [56], choline chloride-SnCl_2_ (1:2) at room temperature for 60–200 min [57] and l-(+)-tartaric acid-dimethyl urea (3:7) at 70 °C for 2 h [58].

On the other hand, organic electrochemistry is considered a clean, atom-economic, and environment-friendly method of synthesis. The electrochemical synthesis relies on the use of electrons as a reactant and energy source, thereby reducing the time and energy needed for the reaction. The method was widely used in the last two decades for the synthesis of organic compounds [59–66]. To date, two publications reported the synthesis of BIMs under electrochemical conditions. The first was published in 2018 by Du and Huang [67] who reported the reaction of indole and ether under electrochemical conditions using LaCl_3_ as a catalyst., The authors utilized platinum as anode and cathode and lithium perchlorate as a supporting electrolyte. The reaction was conducted in THF-acetonitrile at room temperature under 5 mA constant current for 6.5 h. In 2022, Jat et al*.* [68] reported the BIMs synthesis using graphite as anode and cathode in acetonitrile for 90 min. Lithium perchlorate was used as a supporting electrolyte and the reaction was conducted at room temperature under a 10 mA constant current.

**Scheme 1:**
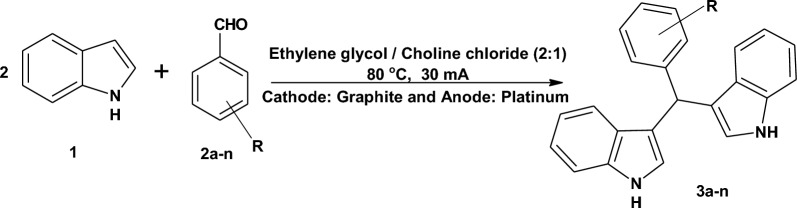
Synthesis of bis(indolyl)methane derivatives **3a-n**

In continuation of our interest in developing new methods of synthesis based on electrochemical reactions [69–72], we reported herein the first synthesis of BIM derivatives under electrochemical conditions in DESs (Scheme 1). The method was characterized by being atom economic and environmentally friendly. The products were obtained in high yields in short reaction time and the reaction tolerated a wide scope of substrates.

## Results and discussion

### Reaction of indole and benzaldehyde in deep eutectic solvents

The reaction of two molar equivalents of indole (**1**) and one molar equivalent of benzaldehyde (**2a**) in different DESs was investigated as a preliminary study. The reaction was conducted by heating at 80 °C in a water bath. The results are presented in Table 1. The best result was obtained upon using ethylene glycol/ choline chloride (2:1) as a solvent with a 50% yield.

**Table 1:** Synthesis of bisindolylmethane **3a** in DESs


Entry	DES	Yield
1	Ethylene glycol/choline chloride (2:1)	50%
2	Propylene glycol/choline chloride (2:1)	Trace
3	Urea/choline chloride (2:1)	NR

All the reactions were conducted at 80 °C for 3h

*NR* no reaction

### Electrochemical reaction of indole and benzaldehyde in deep eutectic solvents

The reaction of two molar equivalents of indole (**1**) and one molar equivalent of benzaldehyde (**2a**) in DESs was studied as a model reaction. The temperature was adjusted to 80 °C to ensure the complete dissolution of the starting materials. Four types of DESs were examined. Choline chloride was mixed with urea, ethylene glycol, propylene glycol, or glycerol in a ratio of 1:2 to form DESs. The reaction was conducted at 10 mA and 30 mA at different times and using different electrode materials. TLC was used to monitor the progress of the reaction. The results are presented in Table 2.

**Table 2:** Synthesis of bisindolylmethane **3a** in DESs using electrochemical conditions


Entry	DES	Time (mins)	Current (mA)	Cathode	Anode	Yield
1	Ethylene glycol/choline chloride (2:1)	15	30	Graphite	Graphite	31%
2	Ethylene glycol/choline chloride (2:1)	30	30	Graphite	Graphite	53%
3	Ethylene glycol/choline chloride (2:1)	45	30	Graphite	Graphite	62%
4	Ethylene glycol/choline chloride (2:1)	60	30	Graphite	Graphite	62%
5	Ethylene glycol/choline chloride (2:1)	90	30	Graphite	Graphite	62%
6	Glycerol/choline chloride (2:1)	45	30	Graphite	Graphite	63%
7	Propylene glycol/choline chloride (2:1)	45	30	Graphite	Graphite	NR
8	Urea/choline chloride (2:1)	45	30	Graphite	Graphite	NR
9	Ethylene glycol/choline chloride (2:1)	60	10	Graphite	Graphite	N.R
10	Ethylene glycol/choline chloride (2:1)	180	10	Graphite	Graphite	53%
11	Ethylene glycol/choline chloride (2:1)	45	30	Platinum	Graphite	40%
12	Ethylene glycol/choline chloride (2:1)	45	30	Graphite	Platinum	71%
13	Ethylene glycol/choline chloride (2:1)	45	30	Copper	Graphite	59%

All the reactions were conducted at 80 °C

*NR* no reaction

The effect of the reaction time was investigated (Entry 1–5). Conducting the reaction in choline chloride/ethylene glycol for 15 min., 30 min. and 45 min. (Entry 1–3) resulted in the production of compound **3a** in yields of 31, 53 and 62%, respectively. Further increase in the reaction time to either 60 min. or 90 min. (Entry 4 and 5) did not affect the product yield. Therefore, the optimum time was chosen as 45 min. for subsequent experiments.

Next, the effect of varying the type of DES was examined. The results indicated that both ethylene glycol and glycerol gave comparable yields of bisindolyl methane **3a** (Entry 3 and 6). However, the reaction was unsuccessful upon using choline chloride/urea or choline chloride/propylene glycol (Entry 7 and 8). These results were consistent with the results in Table 1 where DESs formed of urea and propylene glycol were not efficient. Additionally, the yield obtained under electrochemical conditions was higher (62%) than that obtained by the heating method only (50%) and was obtained in a shorter reaction time (45 min) than the heating method (3 h). As provided in our previous work [69, 72], the use of DESs eliminated the need for supporting electrolytes and shortened the reaction time owing to their catalytic power.

Changing the constant current used from 30 to 10 mA resulted in a lower yield and as expected the reaction needed a longer time to be completed (3 h) (Entry 9, 10).

Finally, the effect of electrode material was investigated. Platinum, copper, and graphite were used as electrodes. The material of the electrode and counter electrode may affect the yield, but the choice of the electrodes was empirical. Therefore, the reaction was performed using different electrodes to optimize the yield of the reaction. The highest yield (71%) was obtained upon using graphite as cathode and platinum as anode (Entry 12). Replacing the graphite cathode with platinum or copper resulted in a decrease in the yield with percentages of 40% and 59%, respectively (Entry 11 and 13). It was noteworthy that the reaction was initiated by anodic oxidation of the indole ring as evidenced by the cyclic voltammetry studies.

The optimum reaction conditions were conducting the reaction at 80 °C at a constant current of 30 mA for 45 min in ethylene glycol/ choline chloride (2:1) deep eutectic solvent in an undivided cell fitted with platinum as anode and graphite as cathode (Entry 12).

### Electrochemical synthesis of bisindolylmethane derivatives 3a-n in deep eutectic solvents

The scope of the reaction was investigated using different types of aromatic aldehydes and the results are presented in Table 3. The reactions were conducted using the optimized conditions; Entry 12 in Table 2. All the substituted aldehydes examined afforded higher yields (82–100%) than benzaldehyde (71%). Substitution of the benzaldehyde with electron donating or electron withdrawing groups gave high yields of the products. The heterocyclic indole-3-carbaldehyde gave 82% yield. Aldehydes susceptible to electrochemical oxidation (hydroxybenzaldehydes) or reduction (nitrobenzaldydes) were also reactive and provided products in high yields.

**Table 3:** Electrochemical synthesis of bisindolylmethane derivatives **3a–n** using the optimum reaction conditions


Entry	Cpd	Ar	Yield (%)	MP (^o^C)	References
1	**3a**	C_6_H_5_	71	116–118	[73]
2	**3b**	4-ClC_6_H_4_	92	107–108	[74]
3	**3c**	4-BrC_6_H_4_	100	108–110	[75]
4	**3d**	4-FC_6_H_4_	92	130–132	[76]
5	**3e**	3-OHC_6_H_4_	60	130–132	[77]
6	**3f**	4-OHC_6_H_4_	93	125–127	[78]
7	**3g**	4-CH_3_C_6_H_4_	83	138–140	[79]
8	**3h**	4-CH_3_OC_6_H_4_	92	130–132	[79]
9	**3i**	3,4-(CH_3_O)_2_C_6_H_3_	94	140–142	[80]
10	**3j**	3,4,5-(CH_3_O)_3_C_6_H_2_	95	141–143	[81]
11	**3k**	4-OH-3-CH_3_OC_6_H_3_	100	121–123	[82]
12	**3l**	2-NO_2_C_6_H_4_	86	125–127	[80]
13	**3m**	4-NO_2_C_6_H_4_	89	190–192	[83]
14	**3n**	3-indolyl	82	198–200	[84]

Reaction conditions: **1** (2 mmol), **2a–n** (1 mmol), in ethylene glycol/choline chloride (2:1; 10 mL) for 45 min. at 80 °C and c.c 30 mA. Graphite and platinum were used as cathode and anode, respectively

Compound **3c** was prepared on a gram scale. The reaction was completed in 90 min resulting in 98.5% yield.

The ^1^H NMR spectra revealed the appearance of the methane CH proton at δ 4.77–6.05 ppm and the two NH protons at δ 7.86–10.87 ppm. While the ^13^C NMR spectra revealed the appearance of the methine carbon at δ 31.38–40.58 ppm.

A comparison between the electrochemical methods reported in the literature and the present method is illustrated in Table 4. The data presented in Table 4 proved the efficiency of the present method in which no catalyst or electrolyte was needed and the reaction was completed in a shorter time. The electrochemical method developed by Jat et al*.* [68] used lithium perchlorate as electrolyte which is expensive, hazardous and toxic. Whilst, the present study relied on the use of the deep eutectic solvent ethylene glycol / choline chloride which is ecofriendly, more economic and served as a solvent and a supporting electrolyte.

**Table 4:** The different electrochemical conditions used for the synthesis of bisindolylmethanes

	Reactants	solvent	Electrolyte	Time	Temp	Cathode	Anode	Current (mA)	Yield (%)	References
1	Indole and ether	THF-acetonitrile	LiClO_4_/LaCl_3_ as catalyst	6.5 h	RT	Platinum	Platinum	5	29–92	[67]
2	Indole and aldehyde	Acetonitrile	LiClO_4_	1.5 h	RT	Graphite	Graphite	10	49–95	[68]
3	Indole and aldehyde	Ethylne glycol/choline chloride	none	45 min	80 °C	Graphite	Platinum	30	60–100	Present work

### Investigation of the reaction mechanism

**Fig. 2:**
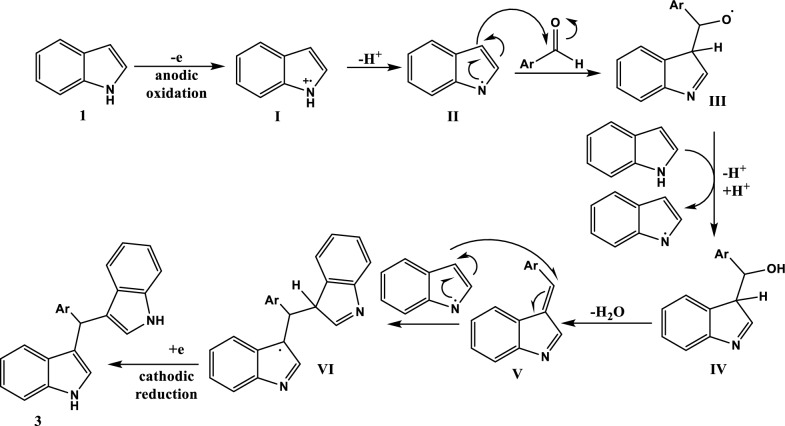
The proposed reaction mechanism for the formation of bisindolylmethane **3a** under electrochemical conditions

The reaction started with anodic oxidation of the indole ring to give a radical cation **I** followed by a loss of a proton to afford the radical **II**. The latter attacked the carbonyl of the aldehyde molecule. This resulted in the formation of radical **III** which oxidized another molecule of indole forming another molecule of radical **II**; while radical **III** was reduced to the intermediate **IV** followed by the elimination of a water molecule. The intermediate **IV** reacted with another indole radical **II** forming radical **VI** that finally underwent a cathodic reduction to afford the final product **3** (Fig. 2).

**Fig. 3:**
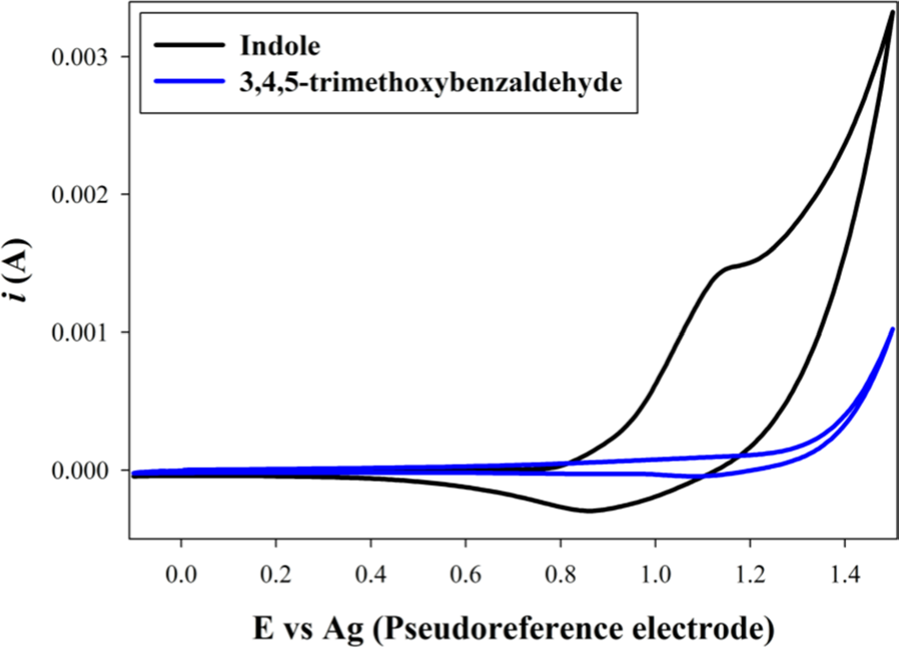
The cyclic voltammograms of 10mM indole and 3,4,5-trimethoxybenzaldehyde in DES at PGE surface vs Ag pseudoreference electrode

The cyclic voltammetry study was consistent with this postulation. Figure 3 showed the cyclic voltammograms of both 10 mM indole, and 10 mM 3,4,5-trimethoxybenzaldehyde at pencil graphite electrode in DES between -0.1 to 1.5 V vs Ag pseudo reference electrode at scan rate 20 mV/s. The results showed a prominent oxidation peak for indole, which started around 0.8 V, while 3,4,5-trimethoxybenzaldehyde showed initiation of oxidation at 1.3 V. These results confirmed that the reaction started by oxidation of indole to produce a cation radical that reacted with the aldehyde to produce the product.

### Experimental and methods

Chemicals were purchased from Sigma-Aldrich, USA. The reaction progress was monitored by TLC using precoated aluminum sheet silica gel MERCK 60F 254 (Merck, Germany) and was visualized by UV lamp. The solvent system used was dichloromethane:ethanol [9:1]. Melting points were obtained using Stuart electrothermal melting point apparatus SMP10 and were uncorrected. ^1^H NMR spectra were conducted using a Bruker Advance 400 MHz NMR spectrometer. ^13^C NMR spectra were performed using a Bruker Advance 100 MHz spectrometer. Chemical shifts were recorded in ppm on a δ scale using DMSO-*d*_6_ as a solvent and coupling constants were recorded (*J*) in Hz. Element analyses were carried out at the Regional Center for Mycology and Biotechnology, Faculty of Pharmacy, Al Azhar University, Egypt.

### Preparation of deep eutectic solvents DESs

Choline chloride was mixed with either ethylene glycol, propylene glycol, glycerol, or urea with a ratio of 1:2, respectively, and heated in a water bath at 80 °C till a homogenous liquid mixture was obtained [69].

### Synthesis of compound 3a in DESs

A mixture of indole (**1**, 2 mmol) and benzaldehyde (1 mmol) in the appropriate DES was reacted at 80 °C for 3h as presented in Table 1. The reaction was traced using TLC. After the completion of the reaction, the mixture was poured into distilled water (50 mL), and the solid formed was filtered and recrystallized from an ethanol–water mixture. The results are reported in Table 1.

### Electrochemical synthesis and optimization of compound 3a in DESs

A mixture of indole (**1**, 2 mmol) and benzaldehyde (1 mmol) in the appropriate DES was reacted in an undivided cell using two variable electrodes at 80 °C for a variable amount of time as presented in Table 2. The reaction was traced using TLC till the reactants' spots disappeared and a new spot for the product appeared. After the completion of the reaction, the mixture was poured into distilled water (50 mL), and the solid formed was filtered and recrystallized from an ethanol–water mixture. The results are reported in Table 2.

### Electrochemical synthesis of bisindolyl methanes 3a-n in DESs

A mixture of indole (**1**, 2 mmol) and the appropriate aldehyde **2a–n** (1 mmol) was dissolved in the deep eutectic solvent composed of ethylene glycol and choline chloride with a ratio of 2:1 (10 mL) at 80 °C. The mixture was exposed to an electric current of 30 mA in an undivided cell for 45 min using a platinum anode and a graphite cathode. The reaction was monitored using TLC. The mixture was poured into distilled water (50 mL), and the solid formed was filtered and recrystallized from an ethanol–water mixture to afford compounds **3a–n**. The results were reported in Table 3.

### Spectral data of bisindolyl methanes 3a–n

#### 3'-(Phenylmethylene)bis(1*H*-indole) (3a)

Light pink solid; IR: 3414 (NH); ^1^H NMR (400 MHz, DMSO-*d*_6_): δ 5.83 (s, 1H, CH), 6.82–6.87 (m, 4H, ArH), 7.01–7.05 (t, 2H, ArH, *J* = 8 Hz), 7.15–7.19 (t, 1H, ArH, *J* = 8 Hz), 7.25–7.28 (t, 4H, ArH, *J* = 8 Hz), 7.33–7.37 (t, 4H, ArH, *J* = 8 Hz), 10.81 (s, 2H, NH, D_2_O exchangeable); ^13^C NMR (100 MHz, DMSO-*d*_6_): δ 39.3, 111.9, 118.5, 118.6, 119.5, 121.3, 123.9, 126.2, 127.0, 128.4, 128.7, 137.0, 145.4; Anal. Calcd for C_23_H_18_N_2_ (322.40): C: 85.68, H: 5.63, N: 8.69. Anal Found: C: 85.51, H: 5.80, N: 8.95.

#### 3,3′-((4-Chlorophenyl)methylene)bis(1*H*-indole) (3b)

Light pink solid; IR: 3410 (NH); ^1^H NMR (400 MHz, DMSO-*d*_6_): δ 5.86 (s, 1H, CH), 6.83–6.89 (m, 4H, ArH), 7.02–7.06 (t, 2H, ArH, *J* = 8 Hz), 7.26–7.28 (d, 2H, ArH, *J* = 8 Hz), 7.31–7.33 (m, 3H, ArH), 7.35–7.37 (m, 3H, ArH), 10.87 (s, 2H, NH, D_2_O exchangeable); ^13^C NMR (100 MHz, DMSO-*d*_6_): δ 39.3, 112.0, 118.0, 118.7, 119.5, 121.4, 124.0, 126.9, 128.4, 130.5, 130.7, 137.0, 144.4; Anal. Calcd for C_23_H_17_ClN_2_ (356.85): C: 77.41, H: 4.80, N: 7.85. Anal Found: C: 77.63, H: 5.01, N: 8.12.

#### 3,3′-((4-Bromophenyl)methylene)bis(1*H*-indole) (3c)

Buff solid; IR: 3406 (NH); ^1^H NMR (400 MHz, DMSO-*d*_6_): δ 5.83 (s, 1H, CH), 6.83–6.85 (m, 2H, ArH), 6.87–6.89 (t, 2H, ArH, *J* = 8 Hz), 7.02–7.06 (t, 2H, ArH, J = 8 Hz), 7.26–7.31 (m, 4H, ArH), 7.34–7.36 (d, 2H, ArH, *J* = 8 Hz), 7.44–7.46 (d, 2H, ArH, J = 8 Hz), 10.87 (s, 2H, NH, D_2_O exchangeable); ^13^C NMR (100 MHz, DMSO-*d*_6_): δ 39.5, 112.0, 117.9, 118.7, 119.2, 119.4, 121.4, 124.1, 136.9, 131.0, 131.3, 137.0, 144.9; Anal. Calcd for C_23_H_17_BrN_2_ (401.30): C: 68.84, H: 4.27, N, 6.98. Anal Found C: 69.05, H: 4.43, N: 7.16.

#### 3,3′-((4-Fluorophenyl)methylene)bis(1*H*-indole) (3d)

Buff solid; IR: 3410 (NH); ^1^H NMR (400 MHz, CDCl_3_): δ 5.79 (s, 1H, CH), 6.55—6.56 (m, 2H, ArH), 6.85–6.95 ( m, 4H, ArH), 7.07–7.11 (t, 2H, ArH, *J* = 8 Hz), 7.17– 7.22 (m, 2H, ArH), 7.26—7.29 (m, 4H, ArH), 7.84 ( s, 2H, NH, D_2_O exchangeable); ^13^C NMR (100 MHz, CDCl_3_): δ 39.4, 111.1, 114.8, 115.0, 119.3, 119.5, 119.8, 122.0, 123.6, 126.9, 130.1, 136.7, 139.7; Anal. Calcd for C_23_H_17_FN_2_ (340.39): C: 81.16, H: 5.03, N: 8.23. Anal Found C: 80.98, H: 5.21, N: 8.50.

#### 3-[Di(1*H*-indol-3-yl)methyl]phenol (3e)

Pink solid; IR: 3500–3300 (OH), 3410 (NH); ^1^H NMR (400 MHz, DMSO-*d*_6_): δ, 5.86 (s, 1H, CH), 6.70 (s, 1H, OH, D_2_O exchangeable), 6.70–6.73 (m, 3H, ArH), 6.80–6.81 (m, 1H, ArH), 6.97–7.01 (m, 2H, ArH), 7.03–7.05 (m, 1H, ArH), 7.16–7.21 (m, 3H, ArH), 7.37–7.39 (m, 2H, ArH), 7.41–7.43 (m, 2H, ArH), 7.94 (s, 2H, NH, D_2_O exchangeable); ^13^C NMR (100 MHz, DMSO-*d*_6_): δ 39.9, 111.0, 113.1, 115.6, 119.2, 119.9, 121.4, 121.9, 123.6, 127.0, 129.4, 130.9, 136.6, 146.0, 155.4; Anal. Calcd for C_23_H_18_N_2_O (338.14) C: 81.63, H: 5.36, N: 8.28. Anal Found C: 81.49, H: 5.53, N: 8.40.

#### 4-[Di(1*H*-indol-3-yl)methyl]phenol (3f)

Purple solid; IR: 3500–3200 (OH), 3406 (NH); ^1^H NMR (400 MHz, DMSO-*d*_6_): δ 5.85 ( s, 1H, CH), 6.67 (s, 1H, OH, D_2_O exchangeable), 6.75 (d, 2H, ArH, J = 8 Hz), 7.03 (t, 2H, ArH, J = 8 Hz), 7.17—7.24 (m, 5H, ArH), 7.37—7.42 (m, 5H, ArH), 7.93 (s, 2H, NH, D_2_O exchangeable); ^13^C NMR (100 MHz, DMSO-*d*_6_): δ 39.3, 102.6, 111.0, 115.0, 119.2, 119.9, 121.9, 123.5, 127.0, 129.8, 136.3, 136.7, 153.7; Anal. Calcd for C_23_H_18_N_2_O (338.40) C: 81.63, H: 5.36, N: 8.28. Anal Found: C: 81.50, H: 5.42, N: 8.49.

#### 3,3′-(*p*-Tolylmethylene)bis(1*H*-indole) (3g)

Buff solid; IR: 3410 (NH); ^1^H NMR (400 MHz, DMSO-*d*_6_): δ 2.31 (s, 3H, CH_3_), 5.83 (s, 1H, CH), 6.85—6.86 (m, 2H, ArH), 6.90 (t, 2H, ArH, J = 8 Hz), 7.06—7.13 (m, 5H, ArH), 7.27—7.33 (m, 3H, ArH), 7.39 (d, 2H, ArH, J = 8 Hz), 10.84 (s, 2H, NH, D_2_O exchangeable); ^13^C NMR (100 MHz, DMSO-*d*_6_): δ 21.0, 39.3, 111.8, 118.5, 118.6, 119.5, 121.3, 123.9, 127.0, 128.6, 129.0, 135.0, 137.0, 142.4; Anal. Calcd for C_24_H_20_N_2_ (336.43) C: 85.68, H: 5.99, N: 8.33. Anal Found C: 85.51, H: 6.12, N: 8.54.

#### 3,3′-((4-Methoxyphenyl)methylene)bis(1*H*-indole) (3h)

Buff solid; IR: 3410 (NH); ^1^H NMR (400 MHz, DMSO-*d*_6_): δ 3.71 (s, 3H, CH_3_), 5.77 (s, 1H, CH), 6.79–6.80 (m, 2H, ArH), 6.82–6.87 (m, 4H, ArH), 7.03 (t, 2H, ArH, J = 8Hz), 7.25–7.28 (q, 4H, ArH, J = 6 Hz), 7.34 (d, 2H, ArH, J = 8 Hz), 10.78 (s, 2H, NH, D_2_O exchangeable); ^13^C NMR (100 MHz, DMSO-*d*_6_): δ 39.3, 55.3, 111.8, 113.8, 118.5, 118.8, 119.6, 121.2, 123.8, 127.0, 129.6, 137.0, 137.4, 157.7; Anal. Calcd for C_24_H_20_N_2_O (352.16) C: 81.79, H: 5.72, N: 7.95. Anal Found C: 82.05, H: 5.68, N: 8.12.

#### 3,3′-((3,4-Dimethoxyphenyl)methylene)bis(1*H*-indole) (3i)

Buff solid; IR: 3406 (NH); ^1^H NMR (400 MHz, DMSO-*d*_6_): δ 3.70 (s, 3H, OCH_3_), 3.76 (s, 3H, OCH_3_), 5,82 (s, 1H, CH), 6.86—6.87 (d, 2H, ArH, J = 2 Hz), 6.88–6.90 (m, 2H, ArH), 6.91–6.94 (m, 2H, ArH), 7.05–7.10 (m, 3H, ArH), 7.34 (d, 2H, ArH, J = 8 Hz), 7.39 (d, 2H, ArH, J = 8 Hz), 10.82 (s, 2H, NH, D_2_O exchangeable); ^13^C NMR (100 MHz, DMSO-*d*_6_): δ 39.3, 55.9, 111.8, 112.0, 113.0, 118.5, 118.8, 119.6, 120.6, 121.2, 123.9, 127.1, 137.0, 137.9, 147.3, 148.8; Anal. Calcd for C_25_H_22_N_2_O_2_ (382.45) C: 78.51, H: 5.80, N: 7.32. Anal Found C: 78.69, H: 5.94, N: 7.50.

#### 3,3′-((3,4,5-Trimethoxyphenyl)methylene)bis(1*H*-indole) (3j)

Buff solid, IR: 3360 (NH), 1589 (C=C), ^1^H NMR (400 MHz, DMSO-d_6_): δ 3.74 (s, 6H, two CH_3_), 3.86 (s, 3H, CH_3_), 5.85 (s, 1H, CH), 6.62 (s, 2H, ArH), 6.73 (s, 2H, ArH), 7.04 (t, 2H, ArH, J = 8 Hz), 7.02 (t, 2H, ArH, J = 8 Hz), 7.38 (d, 2H, ArH, J = 8 Hz), 7.44 (d, 2H, ArH, J = 8 Hz), 7.99 (s, 2H, NH, D_2_O exchangeable) ppm. ^13^C NMR (100 MHz, DMSO-d_6_): δ 40.5, 56.0, 60.8, 105.8, 111.0, 119.2, 119.5, 119.8, 121.9, 123.5, 127.0, 136.2, 136.7, 139.8, 152.9 ppm. Anal. Calcd for C_26_H_24_N_2_O_3_ (412.48) C: 75.71, H: 5.86, N: 6.79. Anal Found C: 75.54, H: 6.02, N: 6.85.

#### 4-[Di(1*H*-indol-3-yl)methyl]-2-methoxyphenol (3k)

Purple solid, IR: 3610–3200 (OH), 3406 (NH), 1512 (C=C), ^1^H NMR (400 MHz, DMSO-d_6_): δ 3.69 (s, 3H, CH_3_), 5.74 (s, 1H, CH), 6.59 (s, 1H, ArH), 6.74 (s, 1H, ArH), 6.81 (s, 1H, ArH), 6.91–6.95 (m, 2H, ArH), 7.07–7.14 (m, 4H, ArH), 7.27(s, 1H, OH, D_2_O exchangeable), 7.29—7.34 (m, 4H, ArH), 7.85 (s, 2H, NH D_2_O exchangeable) ppm. ^13^C NMR (100 MHz, DMSO-d_6_): δ 39.9, 55.8, 111.1, 111.5, 114.0, 119.2, 119.93, 119.96, 121.3, 121.9, 123.6, 127.0, 136.1, 136.7, 143.8, 146.4 ppm. Anal. Calcd for C_24_H_20_N_2_O_2_ (368.43) C: 78.24, H: 5.47, N: 7.60. Anal Found C: 78.51 H: 5.69 N: 7.78.

#### 3,3′-((2-Nitrophenyl)methylene)bis(1*H*-indole) (3l)

Buff solid, IR: 3406 (NH); ^1^H NMR (400 MHz, DMSO-*d*_6_): δ 4.77 (s, 1H, CH), 6.69–6.70 (m, 3H, ArH), 7.04 (t, 3H, ArH, J = 8 Hz), 7.20 (t, 3H, ArH, J = 8 Hz), 7.35–7.40 (m, 2H, ArH), 7.42–7.44 (m, 3H, ArH), 8.00 (s, 2H, NH, D2O exchangeable) ppm. ^13^C NMR (100 MHz, DMSO-d_6_): δ 34.8, 111.1, 117.6, 119.5, 119.7, 122.2, 123.8, 124.3, 126.7, 127.2, 131.0, 132.3, 136.6, 138.0, 149.8 ppm. Anal. Calcd for C_23_H_17_N_3_O_2_ (367.40) C: 75.19, H: 4.66, N: 11.44. Anal Found C: 75.43, H: 4.79, N: 11.67.

#### 3,3′-((4-Nitrophenyl)methylene)bis(1*H*-indole) (3m)

Reddish brown solid, IR: 3410 (NH); ^1^H NMR (400 MHz, DMSO-*d*_6_): δ 6.04 (s, 1H, CH), 6.87–6.91 (m, 3H, ArH), 7.06 (t, 3H, ArH, J = 8 Hz), 7.29 (d, 2H, ArH, J = 8 Hz), 7.37 (d, 2H, ArH, J = 8 Hz), 7.62 (d, 2H, ArH, J = 8 Hz), 8.15 (d, 2H, ArH, J = 8 Hz), 10.94 (s, 2H, NH, D_2_O exchangeable) ppm. ^13^C NMR (100 MHz, DMSO-d_6_): δ 39.2, 112.0, 117.1, 118.9, 119.3, 121.6, 123.8, 124.3, 126.8, 129.9, 137.0, 146.2, 153.6 ppm. Anal. Calcd for C_23_H_17_N_3_O_2_ (367.40) C: 75.19, H: 4.66, N: 11.44. Anal Found C: 75.40, H: 4.59, N: 11.63.

#### Tri(1*H*-indol-3-yl)methane (3n)

Buff solid, IR: 3406 (NH); ^1^H NMR (400 MHz, DMSO-*d*_6_): δ 6.05 (s, 1H, CH), 6.85 (t, 3H, ArH, J = 7 Hz), 6.93–6.94 (m, 3H, ArH), 7.01 (t, 3H, ArH, J = 8 Hz), 7.32 (d, 3H, ArH, J = 8 Hz), 7.39 (d, 3H, ArH, J = 8 Hz), 10.71 (s, 3H, NH, D_2_O exchangeable) ppm. ^13^C NMR (100 MHz, DMSO-*d*_6_): δ 31.3, 111.8, 118.4, 118.7, 119.7, 121.1, 123.6, 127.2, 137.0 ppm. Anal. Calcd for C_25_H_19_N_3_ (361.44) C: 83.08, H: 5.30, N: 11.63. Anal Found C: 82.87, H: 5.46, N: 11.85.

### Electrochemical synthesis of compound 3c at gram-scale

A mixture of indole (**1**) (1.26 g, 0.01 mol) and 4-bromobenzladehyde (**2c**) (1 g, 0.005 mol) was suspended in ethylene glycol/choline chloride (2:1; 20 mL) in an undivided cell equipped with graphite as cathode and platinum as anode. The electrochemical reaction was conducted at constant current (30 mA), at 80 °C for 90 min. The reaction was cooled and poured into water (250 mL). The solid formed was filtered, dried, and recrystallized from ethanol. The product was obtained in 98.5% yield (1.28 g).

### Cyclic voltammetry study

#### Experimental setup

The study used the traditional three electrode configuration, where silver and platinum wires were employed as quasi-reference electrode and counter electrode, respectively. While the working electrode was a Pencil graphite electrode (PGE). The solvent was choline chloride/ethylene glycol (1:2) which also acted as a supporting electrolyte. The DES was degassed with N_2_ for 15 min then the study was carried out at 80 °C. The results are presented in Fig. 3.

## Conclusion

In summary, this study focused on developing a new environmental friendly method for the synthesis of 3,3′-bis(indolyl)methanes due to their various pharmaceutical applications. Accordingly, an electrochemical method was developed for the synthesis of bis(indolyl)methanes in deep eutectic solvents in good to excellent yields. The use of deep eutectic solvents proved to be easy to use, affordable, and environmentally friendly. Moreover, the use of deep eutectic solvents eliminated the need for another electrolyte or catalyst which fulfilled the atom economy principle for green sustainable chemistry. A series of aromatic aldehydes was reacted with indole successfully using the optimum reaction parameters. The newly developed method was effective across a wide array of substituted aldehydes with different substituents at different positions as well as heterocyclic aldehydes. The reaction mechanism was validated using cyclic voltammetry. Further work are currently ongoing to examine the effect of the prepared compounds as chemosensors for the detection of ions.

## Data Availability

All data that supports the findings of this study is available on request by the authors.
